# Bi-isotype immunoglobulins enhance antibody-mediated neutrophil activity against *Plasmodium falciparum* parasites

**DOI:** 10.3389/fimmu.2024.1360220

**Published:** 2024-04-08

**Authors:** Rodney Ogwang, Lewis Murugu, Irene N. Nkumama, Lydia Nyamako, Oscar Kai, Kennedy Mwai, Linda Murungi, Richard Idro, Philip Bejon, James Tuju, Sam Muchina Kinyanjui, Faith H. A. Osier

**Affiliations:** ^1^ Centre for Geographic Medicine Research (Coast), Kenya Medical Research Institute-Wellcome Trust Research Programme, Kilifi, Kenya; ^2^ College of Health Sciences, Makerere University, Kampala, Uganda; ^3^ Department of Biological Sciences, Pwani University, Kilifi, Kenya; ^4^ Centre of Infectious Diseases, Heidelberg University Hospital, Heidelberg, Germany; ^5^ Centre for Tropical Medicine and Global Health, Nuffield Department of Medicine, University of Oxford, Oxford, United Kingdom; ^6^ Department of Life Sciences, Imperial College London, London, United Kingdom

**Keywords:** malaria, monoclonal antibodies, neutrophils, semi-immune, Kenya

## Abstract

**Background:**

Malaria remains a major global health priority, and monoclonal antibodies (mAbs) are emerging as potential new tools to support efforts to control the disease. Recent data suggest that Fc-dependent mechanisms of immunity are important mediators of protection against the blood stages of the infection, but few studies have investigated this in the context of mAbs. We aimed to isolate mAbs agnostic to cognate antigens that target whole merozoites and simultaneously induce potent neutrophil activity measured by the level of reactive oxygen species (ROS) production using an antibody-dependent respiratory burst (ADRB) assay.

**Methods:**

We used samples from semi-immune adults living in coastal Kenya to isolate mAbs that induce merozoite-specific ADRB activity. We then tested whether modifying the expressed IgG1 isotype to an IgG–IgA Fc region chimera would enhance the level of ADRB activity.

**Results:**

We isolated a panel of nine mAbs with specificity to whole merozoites. mAb J31 induced ADRB activity in a dose-dependent fashion. Compared to IgG1, our modified antibody IgG–IgA bi-isotype induced higher ADRB activity across all concentrations tested. Further, we observed a negative hook effect at high IgG1 mAb concentrations (i.e., >200 µg/mL), but this was reversed by Fc modification. We identified MSP3.5 as the potential cognate target of mAb J31.

**Conclusions:**

We demonstrate an approach to engineer mAbs with enhanced ADRB potency against blood-stage parasites.

## Introduction

Malaria remains a major public health priority. This is particularly true in Sub-Saharan Africa (SSA) where the burden of morbidity and mortality is the highest (1). Naturally acquired immunity (NAI) to *Plasmodium falciparum* is known to develop gradually after multiple malaria episodes (2). In areas with a high intensity of malaria transmission, epidemiological data show that the burden of severe and life-threatening malaria is the highest in early childhood, while uncomplicated malaria dominates in older children and young adults. These individuals remain susceptible to infection and are frequently found to have asymptomatic parasitemia. The acquisition of partial control of parasite growth following multiple episodes of malaria is considered semi-immunity (3, 4). Classical passive transfer studies demonstrated that purified immunoglobulins from such adults living in malaria-endemic areas can result in the reduction of parasitemia and resolution of clinical symptoms in non-immune patients (5). Although this evidences the existence of NAI, neither the specificity of the antibodies nor their mechanisms of action have been fully characterized or understood.

Monoclonal antibodies (mAbs) are emerging as potential and exciting new tools to support efforts to control malaria (6–9). Initial concerns about their widespread use in resource-constrained environments in SSA are increasingly being countered by emerging advances in technology that are leading to reduced costs (6), longer serum half-lives, and increased potency (6). Indeed, recent studies have shown that mAbs such as CIS43LS (8, 10) and L9LS (11) that target different epitopes of the *P. falciparum* circumsporozoite protein (PfCSP) show up to 80% protection in controlled human infection studies in naïve adults (8, 10, 11). Moreover, CIS43LS protected adults over a 6-month period spanning an entire malaria transmission season in Mali (8). However, all mAbs currently undergoing field evaluation target sporozoites, while those against merozoites in the blood stage of the parasite life cycle have not progressed to clinical development.

Blood-stage parasites are responsible for the clinical manifestations of malaria. Thus, the development of mAbs against merozoites may be useful for the treatment and prevention of malaria in infants, children, pregnant women, or naïve travelers visiting areas with ongoing malaria transmission. To date, mAbs targeting blood-stage parasites have shown low potency *in vitro* and thus require high concentrations of antibodies (12–14). For example, an approximate serum concentration of 100 mg/kg of reticulocyte-binding protein homolog 5 (PfRH-5)-specific antibody was thought to be necessary for protection from clinical episodes of malaria (15, 16). We hypothesized that this may be because mAbs targeting this life cycle stage were designed with a focus on antibody Fab-mediated neutralization as captured in the growth inhibition assay (GIA) and not on Fc-mediated effector functions that are increasingly understood to be important for immunity (17–22). The GIA measures the capability of antibodies in the absence of immune cells or complement to inhibit parasite replication *in vitro* (23–26). It is widely used as the reference assay for measuring the inhibitory activity of antibodies against blood-stage parasites. However, the correlation between GIA and vaccine efficacy has been relatively inconsistent (27–29). In addition, in studies investigating naturally acquired immunity using the human challenge model, GIA was not correlated with outcome (20). Therefore, a focus on mAbs that induce Fc-mediated function may be a powerful approach to improve their potency.

Neutrophils are the most abundant immune cell type in blood and are effective antimicrobial cells that play a vital role within the innate arm of the immune system (30). Indeed, it has been shown that individuals with lower neutrophil blood counts are predisposed to overwhelming infections (31, 32). In malaria, neutrophils may mediate protection via parasite clearance through phagocytosis (33, 34), the production of reactive oxygen species (ROS) (22, 35, 36), or the release of neutrophil extracellular traps (NETs) (34). High levels of neutrophil activity measured using an antibody-dependent respiratory burst (ADRB) assay have been associated with protection against malaria in multiple studies in different settings (33, 34). Here, we aimed to isolate mAbs that targeted merozoites and simultaneously induced antibody-dependent neutrophil function. We then tested whether this ADRB activity would be enhanced through an IgG–IgA bi-isotype approach that in theory would result in the engagement of a broader panel of Fc receptors (37–39). We first screened adults from malaria-endemic areas in coastal Kenya to identify individuals with total IgG antibodies that induced high ADRB levels against *P. falciparum* merozoites *in vitro*. We then used B cells from these individuals to isolate merozoite-specific mAbs with demonstrable ADRB activity. Thereafter, we applied a genetic engineering strategy to develop an IgG–IgA bi-isotype antibody and tested whether it enhanced the original ADRB activity.

## Results

### Prioritization of samples for the isolation of functional monoclonal antibodies

**Figure 1:**
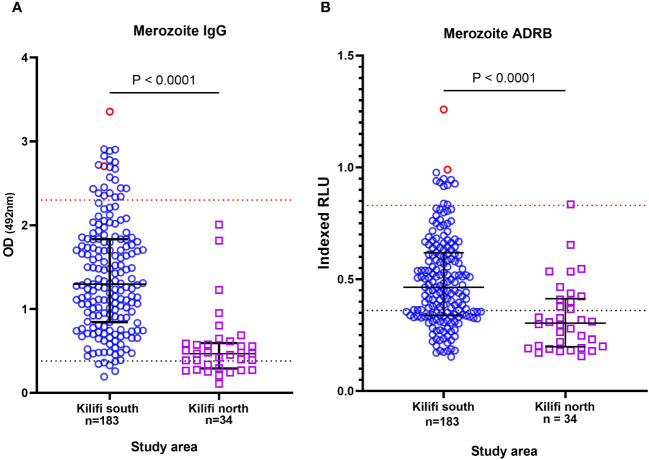
Prioritization of individuals with high levels of functional antibodies. Plot showing the level of **(A)** total anti-merozoite IgG and **(B)** antibody-dependent respiratory burst (ADRB) function. Each point represents an individual from Kilifi South in blue and Kilifi North in purple. The points in red show the individuals selected for monoclonal antibody (mAb) isolation. The blue dotted line represents 3 SD above the mean of negative control (serum from malaria-naïve individual, n = 10). The red line shows the 25th percentile of positive controls (pooled hyperimmune serum).

We began by testing all available serum samples (n = 217) for antibody binding to whole merozoites and the level of ADRB as previously described (19, 21). We then selected samples that had high levels of merozoite-specific antibodies and that induced high levels of ADRB (33, 34). Adults from Kilifi South (an area of moderate-to-high malaria transmission (40)) had significantly higher total merozoite antibodies than those from Kilifi North (an area of low transmission intensity (41)) (p < 0.0001) (**Figure 1A**). This was equally true for antibodies that induced ADRB (**Figure 1B**). There was a strong positive correlation between the level of total merozoite IgG and the level of ADRB targeting merozoites (r = 0.65, p < 0.0001) (**Supplementary Figure 1**). To down-select individuals for mAb isolation, we defined a threshold for high levels of ADRB using our positive control of pooled hyperimmune serum (PHIS). We classified samples with ADRB levels above the 25th percentile of that induced by PHIS as having high ADRB. Ten of 217 (4.6%) samples met these criteria, and we prioritized them for mAb isolation. All 10 samples were from Kilifi South.

### Antigen-agnostic isolation of merozoite-specific mAbs

Next, to generate a panel of merozoite-specific mAbs, an antigen-agnostic, negative selection strategy was employed to isolate memory B cells (CD19^+^/IgG^+^) from cryopreserved peripheral blood mononuclear cells (PBMCs) from two individuals with the highest ADRB activity (**Figure 1B**; **Supplementary Figure 2**). Memory B cells were sorted (4 cells/well) into 384-well plates that contained culture media supplemented with CD40L, IL-2, and IL-21 to enable proliferation and differentiation into antibody-producing cells (42). B-cell differentiation and proliferation were determined by total IgG ELISA of the culture supernatants after 12–13 days of culture. To select for *merozoite*-specific mAbs, supernatants from individual wells were screened using ELISA to whole merozoites of the laboratory-adapted *P. falciparum* 3D7 strain. From one individual, 54 wells of 760 had detectable levels of merozoite-specific IgG within culture supernatant, and of these, 17 wells that had the highest levels of anti-merozoite IgG were prioritized (**Supplementary Figure 3**). From the second individual, of the 464 wells with sorted and cultured cells, 14 wells showed low-level merozoite-specific IgG. Next, mRNA was extracted from B cells in the 31 selected wells expressing merozoite-specific IgG and cDNA synthesized. Thereafter, a published nested PCR protocol (43) was used to amplify the IgG variable region corresponding to heavy and light chain genes from the cDNA of the selected wells (**Supplementary Figure 4A**) (43). These were subsequently cloned upstream of the human IgG1 expression vectors and co-transfected into Expi293F cells. A total of 35 human mAbs were successfully expressed as shown by dot blots detecting human IgG following expression (**Supplementary Figure 4B**).

**Figure 2:**
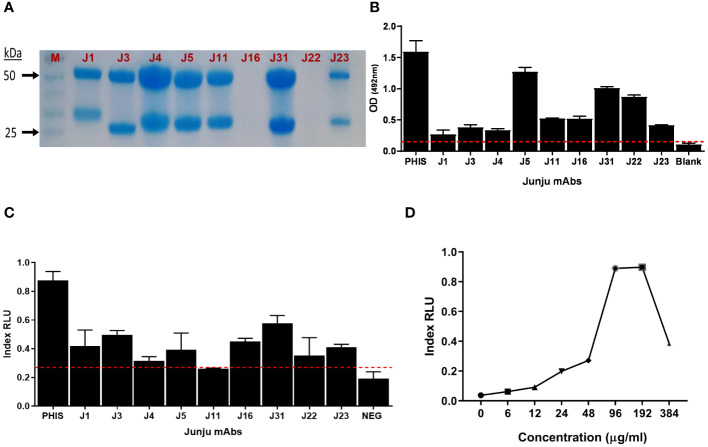
Isolation of merozoite-specific monoclonal antibodies (mAbs) with antibody-dependent respiratory burst (ADRB) activity. **(A)** Sodium dodecyl sulfate (SDS) gel showing nine selected mAbs. **(B)** The level of mAb reactivity to whole merozoites by ELISA. **(C)** The level of ADRB activity for the different mAbs. Error bars represent the interquartile range of two replicates tested at 100 µg/mL. The red line shows seropositivity cut-off of 3 SD above the mean of three negative control replicates. **(D)** Dose–response curve of the level of ADRB activity for mAb Junju 31. Each point represents a single replicate for antibody at each concentration.

We then tested mAb binding to whole merozoites and observed that 9/35 (25.7%) bound to merozoites, which are shown in **Supplementary Figure 4C**. These nine mAbs showed variable levels of expression (**Figure 2A**), reactivity to whole merozoites (**Figure 2B**), and ADRB activity (**Figure 2C**). We selected mAb J31 for further characterization because it induced the highest relative ADRB activity with expression yields at approximately 193 µg/mL of culture. For mAb J31, ADRB activity increased in a dose-dependent pattern with an increase in concentration, although we observed a hook effect (a phenomenon where the effectiveness of antibody is decreased when antibody or antigen is in excess) at antibody concentrations above ~200 µg/mL (**Figure 2D**).

### The cognate target of mAb J31 is MSP3.5

**Figure 3:**
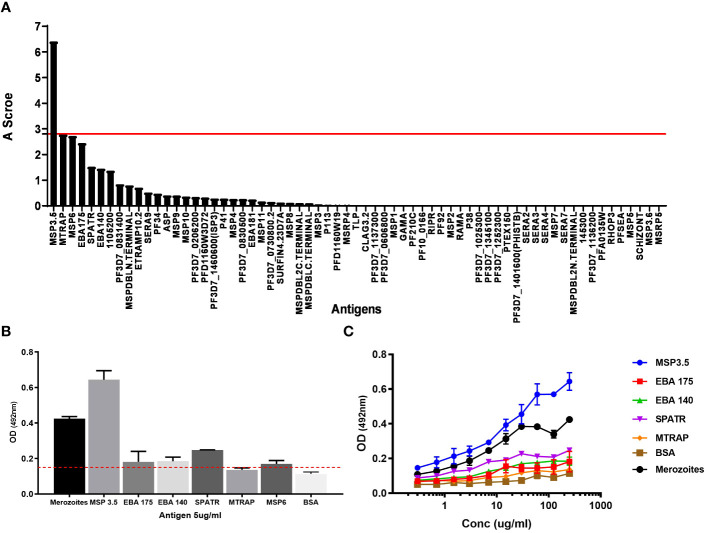
Cognate antigen identification for J31. **(A)** The affinity score of antigens recognized by the monoclonal antibody (mAb) J31 using KILchip microarray. The red line shows the positivity cut-off score (A = 2.8) above which significant binding is considered. **(B)** ELISA confirmation of cognate antigen. For ELISA, antigens were coated at 5 μg/mL, and mAb J31 was tested at 100 μg/mL. The red line shows reactivity cut-off of 3 SD above the mean of bovine serum albumin (BSA). **(C)** Serial dilution of mAb J31 to different antigens. MSP6 not included due to antigen availability. In both **(B, C)**, whole merozoite extract was prepared using laboratory-adapted *Plasmodium falciparum* 3D7 stain included as positive control.

Next, to identify the cognate target of mAb J31, we took advantage of our in-house protein microarray (KILchip 1.0), which consists of 111 correctly folded merozoite stage antigens prioritized as potential targets of naturally acquired immunity (44). We optimized the chip assay for mAb binding and determined that using a concentration < 3 µg/mL of mAb was the most appropriate. We applied a published analytical approach to investigate the specificity of J31. This involved creating an A score that represented the number of standard deviations above the background mean fluorescence intensity (MFI) of all antigens on the chip. An A score above 2.8 was considered a significant antibody–antigen interaction (45). We ranked all the A scores from the highest to lowest and found that J31 recognized MSP3.5 with the highest A score (**Figure 3A**). To confirm this, we then evaluated binding to the six antigens with the highest A scores from the microarray analysis by a more stringent ELISA. These were merozoite thrombospondin-related anonymous protein (MTRAP), merozoite surface protein-6 (MSP-6), and erythrocyte binding antigen-175 (EBA-175), erythrocyte binding antigen-140 (EBA-140), and secreted protein with altered thrombospondin repeat (SPATR). MSP3.5 remained the target antigen with the highest OD value (**Figures 3B, C**). Additional analysis using more specific assays, such as surface plasmon resonance (SPR), and with lower concentrations of the mAb J31 and antigen is needed to confirm the specificity of the interaction between mAb J31 and MSP3.5 and not other antigens.

### IgG–IgA “bi-isotype” enhanced mAb ADRB activity

**Figure 4:**
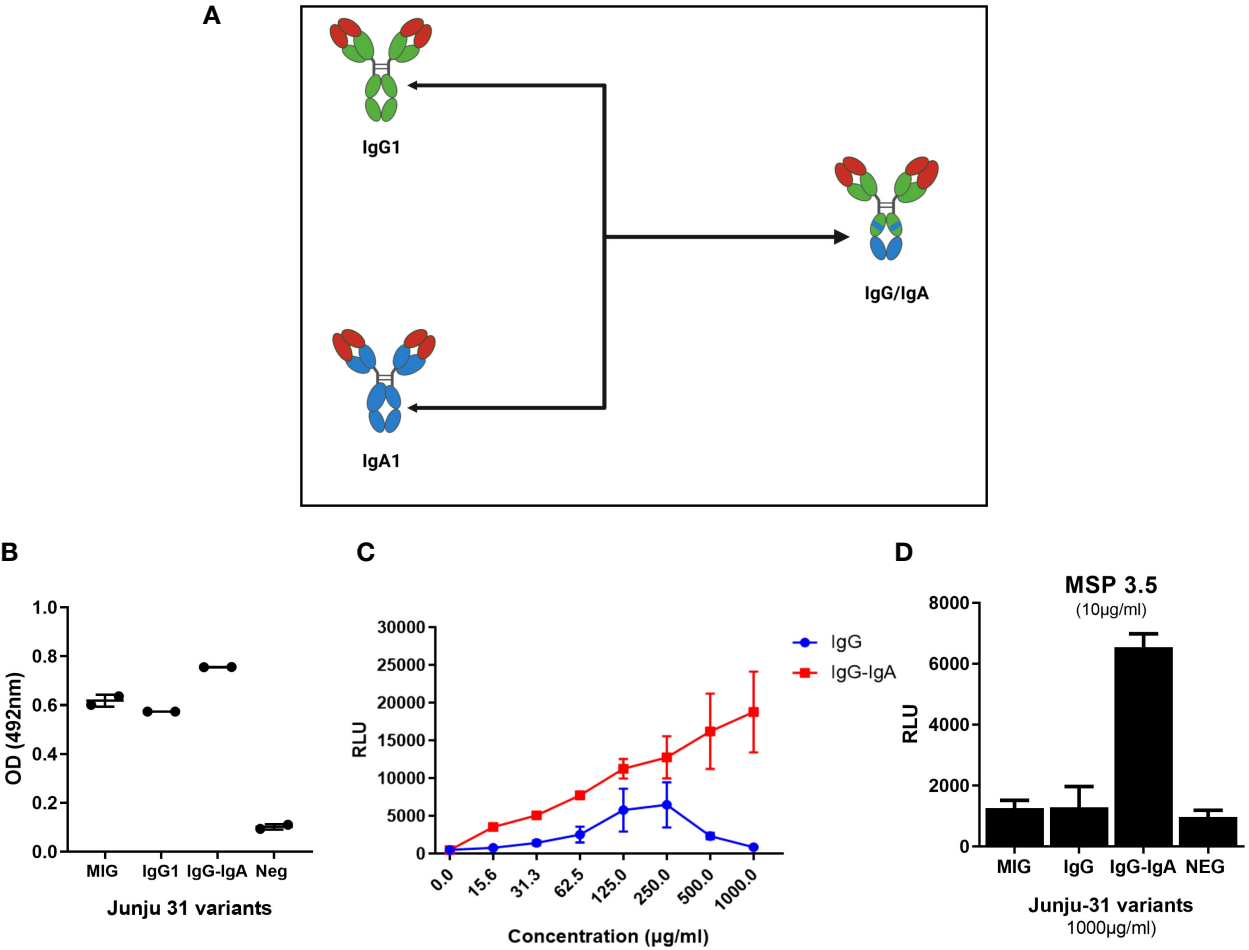
The IgG–IgA “bi-isotype” monoclonal antibody (mAb) enhanced antibody-dependent respiratory burst (ADRB) activity. **(A)** A schematic representation of IgG–IgA showing regions of the Fc carrying IgG1(green) and IgA (blue). **(B)** ELISA showing binding of antibody variants to MSP3.5 coated at 5 μg/mL. **(C)** The level of ADRB activity against whole merozoites of the different variants (red, J31-IgG–IgA; blue, J31-IgG1). **(D)** The level of antigen-specific ADRB activity of the different mAb variants. MSP3.5 was coated at 10 µg/mL, while antibodies were tested at 1 mg/μL.

Next, whether an Fc region cross isotype (IgG–IgA) consisting of IgG and IgA sequences would increase the ADRB activity of J31 was tested (**Figure 4A**). Codon-optimized sequences published by Kelton and colleagues (46) were adopted, our bi-isotype Fc portion was designed, and the construct was ordered from GeneArt. The IgG-IgA Fc region construct was cloned into the heavy chain plasmid to replace the entire IgG1 Fc portion of J31 using restriction sites *Nhe*I and *Dra*III. To confirm successful cloning, a restriction digest with enzyme *Pst*I was conducted and showed three bands for the IgG–IgA heavy chain compared to two bands for the standard IgG1 (**Supplementary Figure 5**). The two mAb variants were then expressed using our standard antibody co-transfection protocol in expi293F cells.

To evaluate binding properties, we showed by ELISA that the engineered IgG–IgA cross isotype recognized MSP3.5 at a comparable level to native IgG1 J31 (**Figure 4B**). Thereafter, we compared the level of ADRB activity between the original and cross-isotype mAb against whole merozoites. The J31 IgG–IgA variant showed a twofold enhanced ADRB activity compared to IgG1. This was consistent across different antibody concentrations, and the level of activity increased in a dose-dependent fashion. Of interest, the hook effect observed with the IgG1 variant at higher antibody doses (**Figures 2D**, **4C**, blue line) was not seen with the IgG–IgA variant (**Figure 4C**, red line). Next, we developed an antigen-specific ADRB assay using MSP3.5. In a similar fashion, IgG–IgA had higher levels of ADRB activity (**Figure 4D**).

## Discussion

Passive transfer of mAbs is emerging as a potential tool that may support the malaria control effort (6, 7). Two mAbs, CIS43LS and L9LS, that target the circumsporozoite protein have shown greater than 80% protection in phase 1 controlled human infection studies (10, 11). Notably, among Malian adults living in an area with intense but highly seasonal malaria transmission, CIS43LS showed a ≤75% reduction in clinical episodes of malaria over 6 months at an intravenous (IV) dose of 10 mg/kg (8). All mAbs that have progressed to clinical evaluation target the sporozoite stages of the parasite life cycle, while mAbs targeting asexual stage merozoites that are responsible for the clinical manifestations of malaria have lagged. Here, we aimed to generate merozoite-specific mAbs with potent Fc-mediated functional activity, as this has been shown to be strongly correlated with protection in recent studies. Using an antigen-agnostic approach, we isolated a panel of functional mAbs targeting whole merozoites. Thereafter, as a proof of principle for *Plasmodium*-specific mAbs, we showed that a bioengineered IgG–IgA cross-isotype mAb can enhance the ADRB activity against merozoites twofold. Similar approaches may now be generalizable to improve the functional potency of future blood-stage mAbs.

Using a similar cross-isotype approach, antibody trastuzumab that targets the Her2 antigen expressed by Her2+ cancer cells was engineered and showed higher Fc-mediated function (46). The mechanism by which the IgG–IgA cross isotype may induce higher levels of ADRB is not completely understood. Our favored hypothesis is that the cross IgG–IgA isotype enhances activation of the immunoreceptor tyrosine-based activation motif (ITAM) in a similar fashion to IgA (39, 47–49). It is thought that since the stoichiometry of the IgA : FcαRI interaction is in a 1:2 ratio, a single cross-isotype mAb would result in the activation of four ITAM molecules, while an IgG : FcγRIIa interaction only engages a single ITAM (47–49). Another possibility is that the IgA : FcαRI interaction is qualitatively superior to IgG : FcγRIIa, resulting in higher activation (48). Important to note is the growing body of evidence that suggests a role for functional IgA antibodies in protection against malaria (50, 51). A post-hoc systems serology analysis within a controlled human infection trial evaluating reticulocyte-binding protein homolog 5 (RH5) showed that the magnitude of anti-RH5 IgA in plasma is associated with a delayed time to malaria diagnosis (50). Importantly, the authors propose that the IgA antibodies may be acting through a neutrophil activation pathway (50). Another independent study demonstrated a protective role for circulating IgA antibodies against pre-erythrocytic parasites (51). These strengthen the possible use case for a potent bi-isotype antibody. The clinical utility of the IgG–IgA cross isotype may be hindered due to the poor serum half-life suggested by low FcγRIIIa/CD16a and FcRn interaction observed with the IgG–IgA–trastuzumab cross isotype (46). However, other chimeric antibody strategies have been developed that may overcome this and potentially allow FcRn interaction. Examples include an anti-CD20 and FcαRI/CD89 IgG bi-specific antibody (37) and chimeras that express either Fcα-Fcγ or Fcγ-Fcα in tandem (38). In addition, despite the fact that mAbs are typically well tolerated, it is important to mention that bi-isotypes may be immunogenic *in vivo* leading to anti-drug antibody (ADA) responses that may lower their efficacy (52). Additional complications include low stability and the potential for adverse reactions such as acute anaphylaxis and serum sickness (53).

Our analysis suggests that MSP3.5 is the cognate target of mAb J31. Additional confirmatory experiments using SPR, immuno-precipitations (54), mass spectrometry (44), and structural studies were considered but not feasible in the prevailing circumstances. This is a major limitation to our antigen identification, which depends on antibody/antigen binding as determined using ELISA. In partial mitigation, we screened our antibody against >100 immunogenic merozoite surface antigens using an established protein microarray platform (44). Importantly, the conclusions made regarding engineering a more functionally active mAb would be supportable irrespective of antigen. MSP3.5 is part of a protein family consisting of eight proteins of which MSP3 (55–59), MSP6 (60, 61), H101, and H103 (62) have been investigated in sero-epidemiological studies and linked with protection against clinical episodes of malaria (63). Notably, two vaccine formulations based on MSP3 have been tested: a long synthetic peptide derived from a conserved region of MSP3 (MSP3-LSP) (64, 65) and a protein fusion consisting of two antigens, GLURP and MSP3 (GMZ2) (66, 67). Although both reported good safety data, they showed poor protective efficacy in phase 1 clinical trials. Members of the family retain a unique motif (a small stretch of amino acids NLRNA/NLRNG) at the N-terminus. However, unlike other members, MSP3.5 does not show high levels of sequence relatedness (≈32% identity and ≈54% similarity of amino acid residues) at the C-terminus (63). MSP3.5 has not yet been studied in the context of naturally acquired immunity or vaccine studies.

Only a limited number of mAbs targeting merozoite antigens have been isolated; however, none have progressed to clinical testing. The level of *in vitro* functions has been investigated primarily using the growth inhibition assay. For example, isolated mAbs targeting the highly conserved RH5 (68) and those targeting the merozoite surface protein 1 complex (MSP1) (69, 70) show >90% growth inhibition but at high concentrations of approximately 1 mg/mL. *In vivo* studies suggest that a dose of 100 mg/kg or higher may be required for clinical protection (15). However, these were modeled using the growth inhibition assay, which may not be the best correlate for protection (16). The development of mAbs to blood-stage antigens faces some challenges like antigenic variation (71, 72) and parasite load, in addition to a very short merozoite extracellular phase (12, 73). These challenges will require careful mAb design and optimization to achieve potent mAbs with clinical relevance. Importantly, our antigen-agnostic approach may increase the likelihood of identifying new antibodies.

Passive transfer studies clearly demonstrated the feasibility of using pooled polyclonal antibodies as a therapy (5, 74). This, therefore, allows optimism that with continued research, the development of potent antibody biologics for malaria treatment is achievable. Importantly, the potential clinical use of mAbs within Africa is becoming plausible with declining costs, improving technology, and growing infrastructure (75, 76). Potential use cases for blood stage-specific mAbs include chemoprophylaxis in regions with highly seasonal malaria transmission (8) and for naïve travelers visiting malaria-endemic regions (77). Additionally, mAbs may be useful for chemoprevention in special populations such as children with sickle cell anemia (78), children recovering from severe malaria who are at increased risk of repeat infection and poor outcomes (79), and pregnant women (80). We did not test the *in vivo* function of our antibodies in murine or non-human primates; therefore, the clinical utility of this mAb requires further investigation. In addition, to strengthen the use case of the cross IgG–IgA bi-isotype, a comparison with IgA variants would be relevant.

## Conclusion

We show that an IgG–IgA cross-type mAb enhances ADRB activity against whole *Plasmodium* merozoites. Similar cross-isotype approaches may be used to enhance the *in vitro* activity of other anti-*Plasmodium* antibodies. Future studies should investigate the *in vivo* utility of this approach describing their efficacy and pharmacokinetic properties. The role of MSP3.5 in naturally acquired immunity should be explored in more detail. Antigen-agnostic approaches may enable the identification of novel vaccine targets.

## Methods

Blood samples were collected from adults participating in studies of naturally acquired immunity to malaria conducted by the KEMRI-Wellcome Trust Research Programme. Samples from which both plasma and PBMCs (n = 217) were available were randomly selected from cross-sectional surveys conducted in 2015, 2017, and 2018. Study participants lived in either Kilifi North or South. These are sublocations within Kilifi County with differing levels of malaria transmission intensity. Kilifi North reports low-level transmission (41), while Kilifi South records moderate-to-high malaria transmission (40).

### Ethical considerations

The samples from coastal Kenya were obtained under ethical approvals granted by the KEMRI Scientific and Ethics Review Unit, approval reference number No. 3149. All participants provided written informed consent. All data were anonymized and stored in secure KEMRI servers in Kilifi, Kenya, and managed by the Kilifi Data Governance Committee.

### Merozoite ELISA

*P. falciparum* merozoites of the 3D7 strains were isolated as described previously (19). Parasites were thawed and cultured to obtain trophozoites at high parasitemia (8%–12%). The trophozoites were isolated by magnetic purification and cultured in a fresh complete medium to allow development to the early schizont stage. A protease inhibitor, *trans*-epoxysuccinyl-l-leucylamido(4-guanidino)butane (E64; Sigma-Aldrich, St. Louis, MO, USA), was added to allow maturation of the schizonts without rupture (81). Merozoites were harvested by filtration of the mature schizonts through 1.2-µm filters (Pall, Port Washington, NY, USA). The merozoites were stained using either 1 µg/mL ethidium bromide (EtBr, Thermo Fisher Scientific, Waltham, MA, USA) or 1× SYBR green dye (Thermo Fisher) for 30 min and counted against CountBright™ Absolute Counting Beads (Thermo Fisher) using BD FACS Canto II flow cytometer.

Next, 96-well plates (Thermo Fisher) were coated with 100 µL/well of 3D7 merozoites at 5 × 10^6^ merozoites/mL at 4°C overnight. The plates were washed and blocked with 200 µL/well of blocking buffer, 1% casein (Thermo Fisher), for 2 hours at 37°C. Samples were diluted at 1:500 with blocking buffer, added at 200 µL/well, and incubated for 1 hour at 37°C. Plates were washed before 100 µL/well of respective secondary antibodies [horseradish peroxidase (HRP)-conjugated: rabbit anti-human IgG (Dako, Glostrup, Denmark)] was added and incubated for 1 hour at 37°C. The plates were then washed four times, and 100 µL/well of the substrate [*o*-phenylenediamine dihydrochloride (OPD); Sigma-Aldrich] was added and incubated for 20 min in the dark at room temperature (RT). The reaction was stopped with 30 µL of 1 M hydrochloric acid (HCl; Sigma-Aldrich), and the absorbance was measured at 492 nM using a BioTek Cytation 3 cell imaging multi-mode reader.

### Antibody-dependent respiratory burst assay

We conducted the ADRB assay as previously described (33, 34) with minor modifications. The assay involves three major steps: i) parasite preparation, ii) neutrophil isolation, and iii) a chemiluminescence detection method.

#### Parasite preparation

*P. falciparum* trophozoites of the 3D7 strain were isolated by magnetic purification and cultured in a fresh complete medium to allow development to the early schizont stage. A protease inhibitor E64 (Sigma-Aldrich) was added to allow the maturation of the trophozoites for 8–12 hours while preventing schizont rupture (81). The schizonts were then quantified and stored at −80°C until use.

At the time of the assay, schizonts (10 × 10^5^ schizonts/mL) or purified antigen (10 µg/mL) in phosphate-buffered saline (PBS) were coated into white opaque 96-well plates (Greiner, Frickenhausen, Germany) at 100 µL/well and incubated overnight at room temperature. The plates were then washed and blocked with 200 μL/well casein in PBS for 1 hour at room temperature. The plates were washed before the addition of 50 μL of diluted plasma (1:50 in 1× PBS) to each well, followed by a 1-hour incubation at 37°C.

#### Neutrophil isolation

Polymorphonuclear leucocytes (PMNs) were prepared from freshly collected whole blood for each assay using a standard procedure. Whole blood at a volume of 40 mL from healthy donors was mixed at a ratio of 1:1 with Hank’s Balanced Salt Solution (HBSS; Thermo Fisher). The diluted blood was carefully layered on 7.5 mL aliquots of Ficoll (GE Healthcare, Chicago, IL, USA). This was then centrifuged at 600 × *g* for 15 min. The supernatant was carefully removed without disturbing the red cell pellet. The pellet was resuspended in 5 mL of HBSS, then mixed with 3% dextran at a ratio of 1:2, and incubated at room temperature in the dark for 30 min. The supernatant was then carefully collected and centrifuged at 500 × *g* for 7 min at 4°C. The supernatant was discarded, and red cell contaminants were lysed. The cells were then centrifuged at 500 × *g* for 7 min at 4°C, and the pellet was resuspended in 1 mL of ice-cold PMN buffer [HBSS with 0.1% bovine serum albumin (BSA; Sigma-Aldrich), 1% d-glucose]. The PMN count was determined using a hemocytometer, and the concentration was adjusted using the PMN buffer to 1.0 × 10^7^/mL. The cells were kept on ice.

#### Chemiluminescence detection

The schizont-coated plates were washed before the addition of 50 μL of PMNs (1 × 10^7^ PMNs/mL) and 50 μL of 0.04 mg/mL isoluminol (Santa Cruz Biotechnology, Dallas, TX, USA) per well. The plates were immediately loaded onto a plate reader, and ADRB activity was quantified via chemiluminescence at 450 nM every 2 min for 1.5 hours, captured as the maximal relative light units (RLUs). The positive controls were PHIS and MIG (Purified human IgG from Malawian adults), while the negative controls were malaria-naïve sera from German donors as described under merozoite ELISAs above. To account for the variability of ADRB activity from PMNs from different individuals, each sample was tested in duplicate using PMNs from two independent donors (22, 36). To further minimize variability between donors, the RLU values were indexed based on the PHIS-positive controls in each plate (22, 36). Thus, the mean RLU for the PHIS control was used as the reference sample with an indexed RLU of 1.0. The indexed RLU value for each test sample was calculated as RLU of sample/RLU of PHIS. The mean indexed RLU values using PMNs from two donors were then calculated.

### Memory B-cell isolation

PBMC samples were thawed and transferred into pre-warmed (37°C) media [Roswell Park Memorial Institute (RPMI) + 50% fetal bovine serum (FBS)]. The tube was then centrifuged at 400 × *g* for 5 min, the supernatant was discarded, and cells were resuspended in cold sterile FACS buffer [3 mL PBS, 2% fetal calf serum (FCS), 2 mM EDTA, and 25 mM HEPES]. Thawed cells were stained for memory B cells with primary fluorophore-conjugated antibodies to human CD3, CD8, CD14, CD19, CD20, CD27, IgG, and IgM (BD Pharmingen, San Diego, CA, USA) in equimolar ratios. Staining was performed for 30 min at 4°C in PBS with 1 mM EDTA and 1% FBS. Cells were selected for the phenotype (CD3^−^/CD8^−^/CD14^−^) while (CD19^+^/CD20^+^). Memory B cells were sorted four cells per well into 384-well plates containing feeder medium [complete Iscove’s Modified Dulbecco’s medium (IMDM), IL-2 (10,000 U/mL), IL-21 (100 μg/mL], and mitomycin C-treated feeder cell (3T3-msCD40L)] and cultured for 13–14 days (42).

### Immunoglobulin gene cloning and generation of recombinant antibodies

From wells with *Plasmodium*-specific B cells of interest, RNA was extracted using TRIzol™ reagent (Thermo Fisher Scientific) following the manufacturer’s instructions with slight modifications. B cells from the select wells were transferred to a separate tube homogenized with TRIzol reagent (1,100 µL), followed by the addition of chloroform (200 µL, Sigma-Aldrich). The homogenate was then centrifuged at 1,400 × *g* for 35 min at 4°C. GlycoBlue 2 µL (Ambion, Austin, TX, USA) was added to a clean RNase-free tube followed by the homogenate aqueous phase and isopropanol (500 µL, Sigma-Aldrich), which were mixed and incubated overnight at 4°C. The next day, samples were centrifuged at 13,000 rpm for 30 min at room temperature, supernatants were discarded, and the pellet was washed gently with 500 µL of 75% ethanol. Thereafter, all ethanol was removed, and the pellet was air-dried and resuspended in RNAsecure (20 µL, Ambion) by heating for 10 min at 60°C. The RNA sample was mixed gently and stored at −80°C.

Gene cloning and recombinant antibody production were performed as previously described (43). Briefly, cDNA was generated from RNA samples using random hexamer primers (SuperScript III First-Strand Synthesis, Invitrogen, Carlsbad, CA, USA). Immunoglobulin (Ig) heavy and corresponding kappa or lambda L-chain gene transcripts were then amplified via semi-nested PCR using primers previously published (43). Amplified IgG variable regions were cloned into human Igh1 [AbVec-hIgKappa (FJ475056)] and Igk [AbVec-hIgG1 (FJ475055)] or Igl [PBR322 based IG-lambda expression vector (FJ517647)] expression vectors. This was conducted using high-fidelity restriction enzymes (New England Biolabs, Ipswich, MA, USA) in CutSmart buffer (AgeI HF, SalI HF for the heavy chain, AgeI-HF, BsiWI-HF for the kappa chain, and AgeI HF, XhoI-HF for the lambda chain). Expression plasmids were transformed into competent cells (One Shot™ Top10 *Escherichia coli*) by heat shock method (82) and plated on Luria–Bertani agar plates containing ampicillin (100 µg/mL). Single colonies were selected and expanded in Luria–Bertani broth containing ampicillin (100 µg/mL) overnight in a shaking incubator (270 rpm, 37°C). In instances where both kappa and lambda light chains were amplified from a single culture well, both variants were cloned and expressed as independent mAbs.

### Antibody expression and purification

The overnight bacterial culture was harvested by centrifugation (6,000 × *g*, 4°C) for 15 min. Expression plasmid DNA was isolated by maxiprep purification following the manufacturer’s instructions (QIAGEN, Valencia, CA, USA) and thereafter used for antibody expression in HEK293F cells using the Expi293 Expression System (Invitrogen) according to the manufacturer’s instructions. Briefly, cells were cultured to a concentration of 3.0 × 10^6^ cells/mL and co-transfected with the appropriate pair of antibody expression vectors using the ExpiFectamine 293 transfection reagent (Invitrogen). Cells were then incubated at 37°C with 8% CO_2_ in an orbital shaker at 125 rpm, and culture supernatants were harvested 6 days post-transfection (44). Antibodies were then purified using protein G resin (Amintra Protein G Resin, Expedeon, Cambridge, UK) following the manufacturer’s instructions. Briefly, clarified expression media were incubated together with G protein resin overnight at 4°C under slow rotation. The resin was then passed through spin columns (Bio-Rad, Hercules, CA, USA) pre-equilibrated with 1× PBS. The resin was washed twice, and the bound antibody was eluted with 0.1 M glycine, pH 3, and neutralized immediately with 1 M Tris, pH 8.

### Maintaining and treatment of feeder cells 3T3-msCD40L

Frozen feeder cells (3T3-msCD40L) were thawed rapidly in a water bath at 37°C. The 3T3-msCD40L cells were added into the culture medium [IMDM with GlutaMAX (Gibco, Grand Island, NY, USA) made to 5% FBS and 1% penicillin-streptomycin (Gibco)]. Cells were seeded in a T-75 flask at 1.0 × 10.0^5^ cells/mL. Cultures were maintained in an incubator at 37°C, with 5% CO_2_ for 3–4 days until confluency. At confluency, the culture supernatant was removed by settling. The attached cells were washed two times with 10 mL PBS-GLUCOSE (Sigma-Aldrich) and warmed to 37°C. Trypsin-EDTA (0.05% Trypsin and 0.53 mM EDTA-4Na (Gibco)] was added and incubated for 1 min at 37°C 5% CO_2_. To stop the reaction, 10 mL 5% FBS IMDM was added, and by gently tapping, attached cells were loosened, washed, reseeded, and cultured in fresh culture media at 37°C with 5% CO_2._ Cells were then treated with 50 mg/mL mitomycin C (Sigma-Aldrich) at a suspension of approximately 1 × 10^6^ cells/mL in 5% FBS IMDM for 4 hours at 37°C. This incubation was performed in closed tubes to allow agitation within a rotation system. The cells were washed four times to remove all the mitomycin C and resuspended to an appropriate cell count for plating into 384-well plates (42).

### Dot blots

Antibody expression was successfully confirmed using standard blots on a 0.2-µm nitrocellulose membrane. Here, 2 μL of culture supernatants was spotted onto the membrane and air-dried for 10 min. A positive control (pooled malaria hyperimmune sera) and negative control [mock transfection with polyethylenimine (PEI) alone] were also included. Subsequently, the membrane was blocked with 1% (milk powder/PBS) for 1 hour. Following blocking, the membrane was washed 3× with PBST (0.1% Tween-20 in 1× PBS) for 5 min. The membrane was then incubated with anti-human IgG-HRP diluted at 1:5,000 in blocking buffer for 1 hour at room temperature. The membrane was washed 3× using PBST. Meanwhile, the substrate (Novex^®^ ECL Chemiluminescent substrate) was prepared by mixing 2 mL luminol (reagent A) with 2 mL enhancer (reagent B) in the dark. The membrane was then incubated with the substrate for 60 sec, and imaging was conducted using the ChemiDoc XRS+ system (Bio-Rad).

### Sodium dodecyl sulfate–polyacrylamide gel electrophoresis gel analysis

This was carried out as previously described. Briefly, the agarose gel was cast between two plates measuring (16 × 16 × 12 cm) with spacers measuring 1.0 mm. A 4-cm-high stacking gel (acrylamide 6% w/v, pH 6.8) was applied on top of the separating gel (acrylamide 10% w/v, pH 8.8). Subsequently, 10 µL of purified monoclonal antibody samples was mixed with the loading buffer [50 mM Tris-HC1, pH 6.8, containing sodium dodecyl sulfate (SDS) 2% w/v, glycerol 10% v/v, bromophenol blue 0.1% w/v] and heated for 10 min at 100°C. The denatured samples were then pipetted on separate wells on the gel. The rainbow protein ladder was also included in a separate well. Electrophoresis was performed at 100 V for 120 min in a chamber containing the running buffer (Tris-glycine chloride, pH 8.3). The gel was developed using InstantBlue^®^ Coomassie Protein Stain.

### Sanger sequencing and sequence analysis

Variable genes cloned into appropriate vectors were sequenced via Big Dye Terminator Cycle sequencing (Applied Biosystems, Foster City, CA, USA), according to the manufacturer’s recommendation. Briefly, a 10-µL reaction mix was prepared consisting of 0.5 µL BigDye terminator 3.1 ready mix, 1.75 µL 5× BigDye sequencing buffer, 4.75 µL of deionized water, 1 µL of 5 µM concentration sequencing primer, and 2 µL of plasmid diluted to 10 ng/mL. Cycle sequencing consists of the following: initial denaturation at 95°C for 10 sec, amplification of 25 cycles (96°C for 10 sec, annealing at 60°C for 5 sec, elongation at 60°C for 4 min), and final extension of 15°C for 10 min. The sequencing reaction was then purified using ethanol/EDTA precipitation, and capillary sequencing was performed using the 3730 DNA analyzer (Thermo Fisher, USA). Sequence analysis was then conducted with Geneious Prime^®^ 2020.0.3, and IgG gene analyses used IGBLAST [a tool used for Ig and T-cell receptor (TR) V domain sequences].

### Statistical analysis

Data were compiled using Microsoft Excel 2016 before being exported into GraphPad Prism version 6.05 for Windows and R studio (R version 4.1.1). The means of normally distributed data were compared using Student’s t-test as appropriate and presented as mean (SD) with a two-tailed p-value <0.05 considered significant. If data were not normally distributed, non-parametric methods like the Mann–Whitney U test were used and presented as median [interquartile range (IQR)]. All correlations were determined using Spearman’s rank correlation test with significance set at p-value <0.05. Antibody seropositivity was defined as a cut-off above the mean optical density (OD) plus 3 standard deviations of malaria-naïve European sera. Seropositivity for ADRB and opsonic phagocytosis assay (OPA) was defined as a cut-off above the mean RLU plus 3 standard deviations above RLU, respectively, of malaria-naïve European sera. The custom protein microarray KILchip was used to screen potential antigen targets of the mAbs. The microarray quantification software captures all antibody intensity parameters in a pre-prepared template referred to as a “.gal” file and stores the data in a “.gpr/.txt” file (44, 83). The analysis method was adopted from Jeong et al. (45). Briefly, an affinity score (A) indicates the number of standard deviations above background-corrected MFIs, and the specificity score (S) represents the difference between the A score of the two antigens with consecutive “A” scores when scores are ranked from the highest to lowest (S_n =_ A_n_ − A_n+1_). “A” scores were determined as A = (I − M)/σ, where “I” is the mean tag subtracted MFI value for any given spot pair, “M” is the median value for “I” for all spots on the array, and “σ” is the standard deviation for “I”. All antigens with an “A” score above 2.8 were considered significant and selected as potential antigen candidates for ELISA analysis. S-scores greater than 3 over the next listed target were considered statistically significant and indicated antibody specificity (45, 83).

## Data availability statement

The raw data supporting the conclusions of this article will be made available by the authors, without undue reservation.

## Ethics statement

The studies involving humans were approved by KEMRI Scientific and Ethics Review Unit. The studies were conducted in accordance with the local legislation and institutional requirements. The participants provided their written informed consent to participate in this study.

## Author contributions

RO: Writing – Original draft, Conceptualization, Investigation, Visualization, Methodology, Writing – review & editing. LeM: Writing – review & editing, Methodology, Visualization, Investigation. INN: Writing – review & editing, Investigation, Methodology. LN: Writing – review & editing, Methodology, Investigation. OK: Writing – review & editing, Methodology. JT: Conceptualization, Investigation, Methodology, Writing – review & editing. PB: Writing – review & editing. KM: Writing – review & editing, Methodology, Formal analysis. LiM: Writing – review & editing, Investigation, Methodology. RI: Writing – review & editing. SK: Funding acquisition, Supervision, Writing – review & editing. FHAO: Writing – original draft, Funding acquisition, Supervision, Writing – review & editing.
