# Supplementary material for: Bi-isotype immunoglobulins enhance antibody-mediated neutrophil activity against Plasmodium falciparum parasites
Source: Front Immunol. 2024 Apr 8;15:1360220. doi: 10.3389/fimmu.2024.1360220 (PMC11033408; doi:10.3389/fimmu.2024.1360220)
Supplement: Supplementary file 1 [file DataSheet_1.pdf]

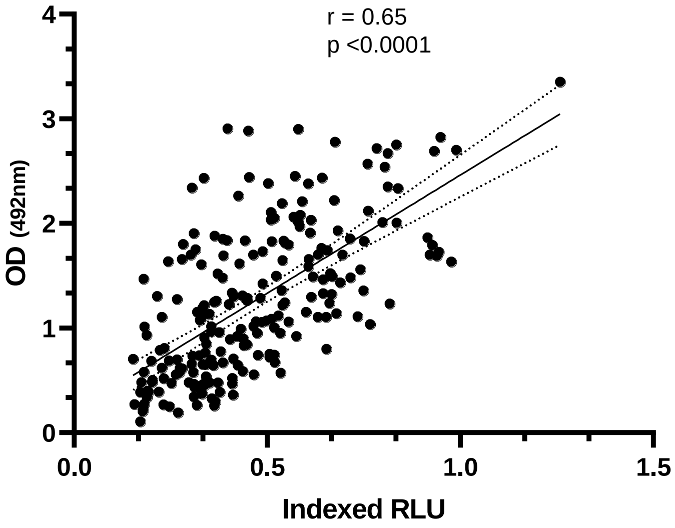

Supplementary figure S1: Scatter plot showing correlation between total merozoite IgG and ADRB.

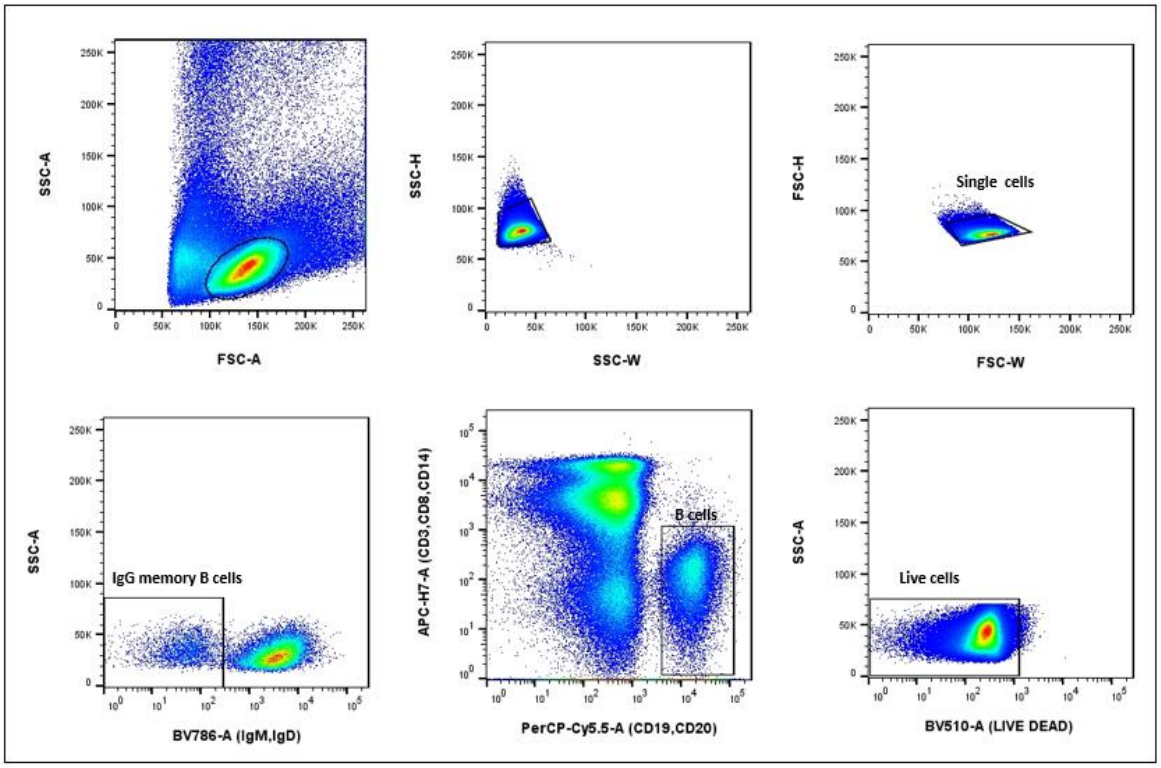

Supplementary figure S2: Representative flow cytometry sort panel to select memory B cells ($CD19^{+}/IgG^{+}$) from cryopreserved PBMCs

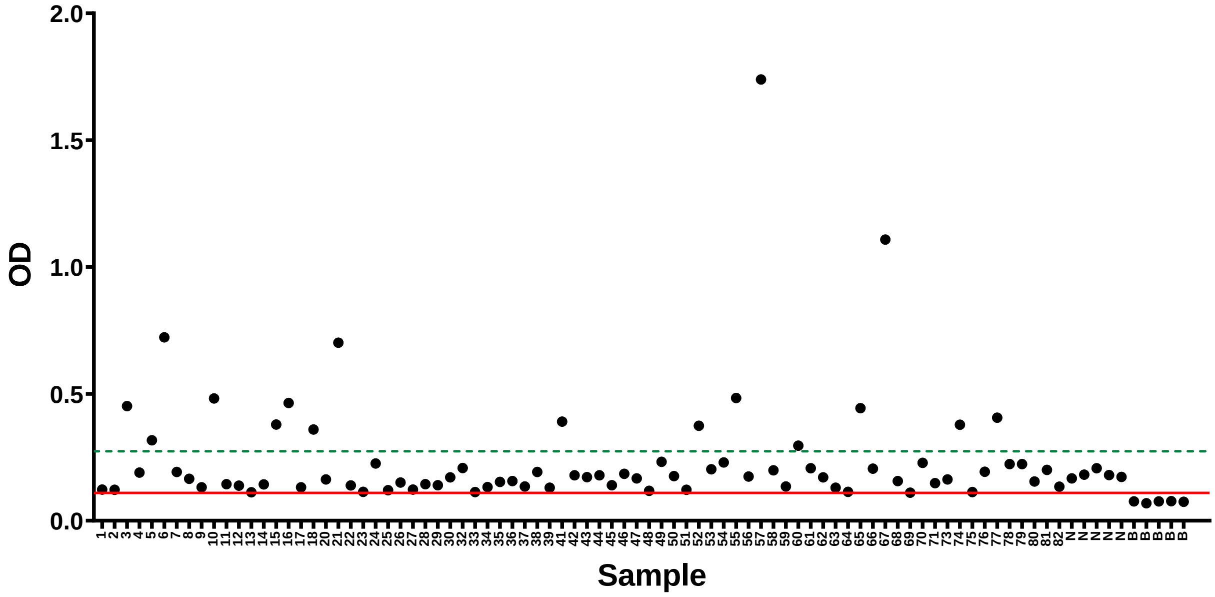

Supplementary figure S3: Selection of wells with proliferated B cells secreting merozoite-specific IgG into supernatants. The red line shows the mean plus 3SD above of blank wells (n = 5) while the green dotted line shows the mean plus 3SD of malaria naïve sera. Representative plot showing tested wells of which 17 with merozoite specific antibodies by ELISA.

B

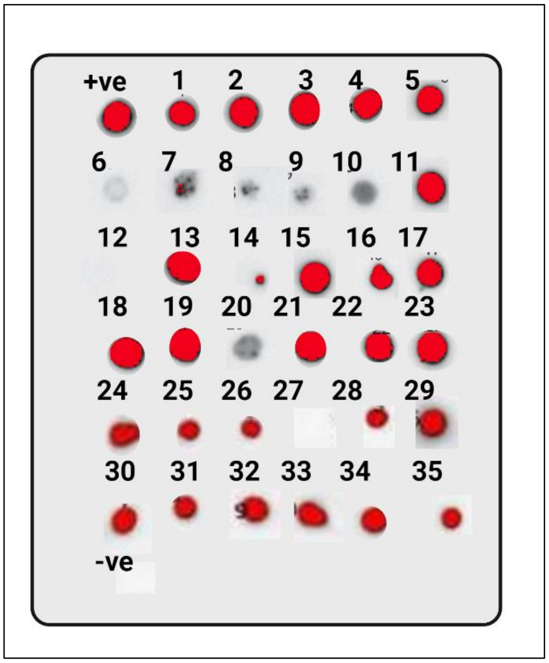

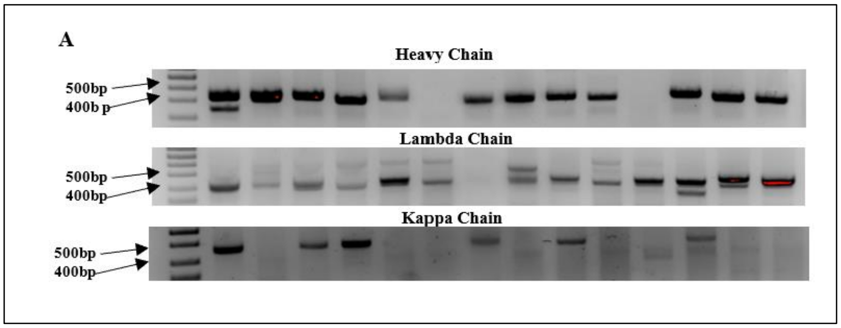

C

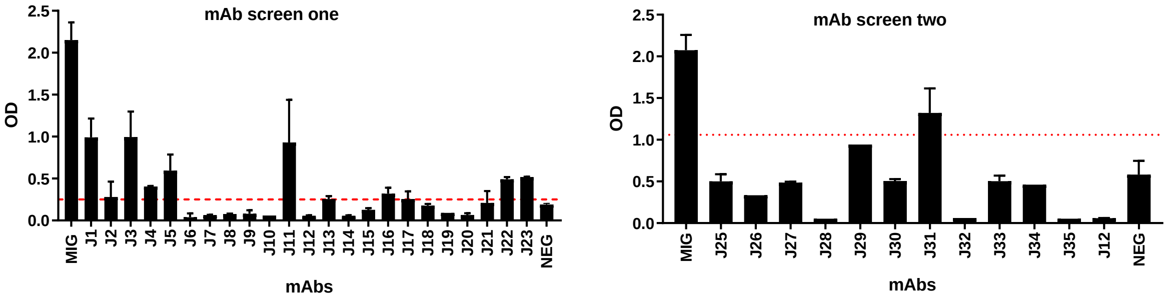

Supplementary figure S4: Generation of monoclonal antibodies **A)** representative gels showing the PCR products of the different variable regions for the heavy chain and the respective lambda and kappa light chains. **B)** Dot bots of the 35 expressed monoclonal antibodies. **C)** screening for merozoite specific mAbs for specificity to merzoites isolated from the laboratory adopted 3D7 parasite line. The red dotted line represents the level of 3SD above the level of negative control (malaria naïve serum) mAbs are labelled with their respective heavy and light chain pairs. **D)** A serial dilution of mAb J31.

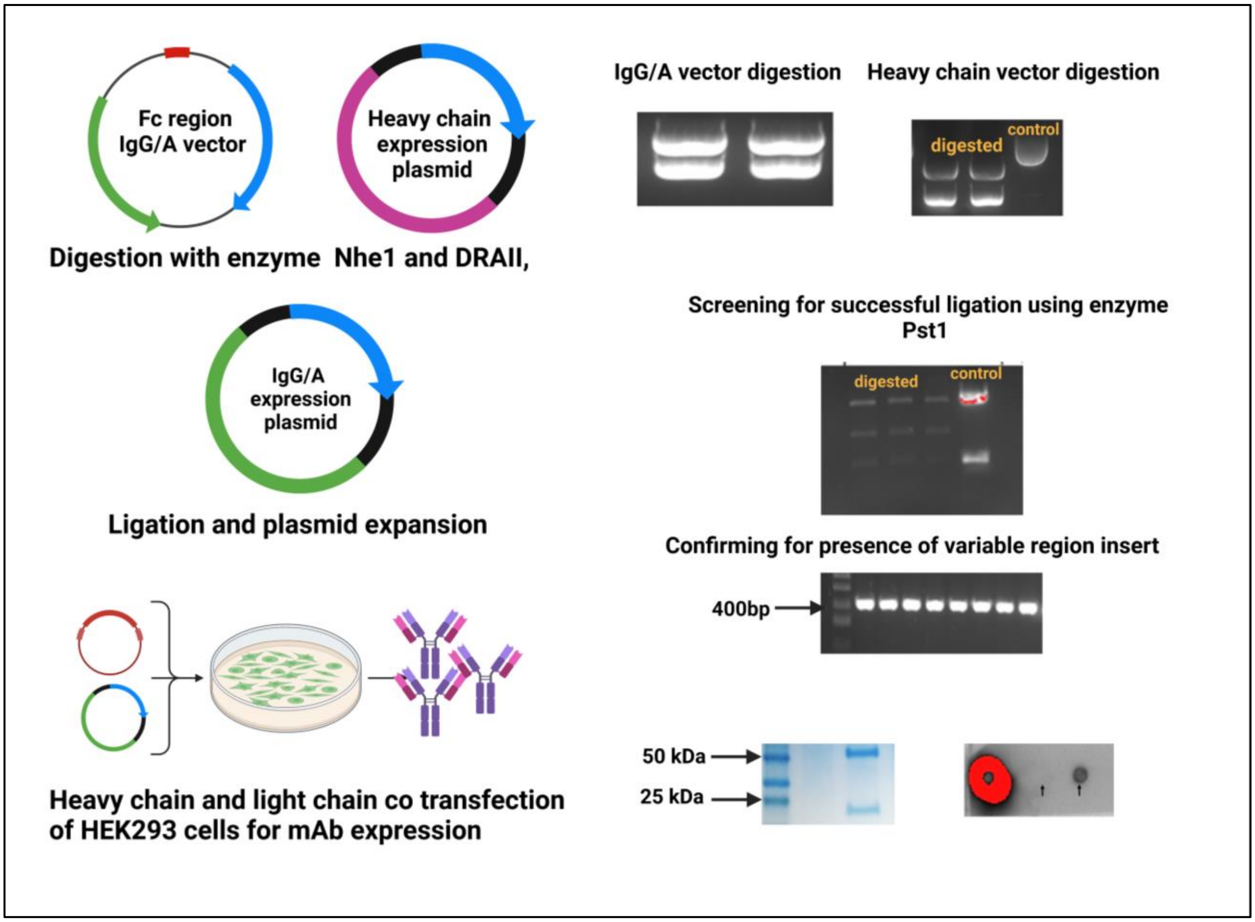

Supplementary figure S5: Schematic showing the development of IgG/A cross isotype antibodies

Supplementary table 1: Germ line usage of the different monoclonal antibodies

mAb	Chain	Allele Usage	Germline similarity (%) to top V gene hit
**J3**	Heavy	IGHV4-59*01, IGHD2-8*02, IGHJ6*04	88.4% (258/292)
	Light	IGLV3-27 IGLJ3*02	92.2% (247/268)
**J7**	Heavy	IGHV3-15*01, IGHD1-26*01, IGHJ5*02	80.3% (240/299
	Light	IGLV1-44*01 IGLJ3*02	93.5% (275/294)
**J13**	Heavy	IGHV5-51*03 IGHD2-21*01 IGHJ1*01	91.4% (265/290)
		IGLV9-49*01 IGLJ3*02	88.0% (271/308)
**J17**	Heavy	IGHV4-61*02, IGHD2-8*01 IGHJ1*01	87.9% (262/298)
	light	IGLV1-47*01 IGLJ3*02	90.5% (266/294)
**J24**	Heavy	IGHV4-4*07 IGHD1-7*01 IGHJ2*01	86.3% (253/293)
	light	IGLV3-21*02 IGLJ2*01	99.0% (285/288)
**J27**	Heavy	IGHV4-34*01 IGHD2-2*02 IGHJ6*03	94.5% (277/293)
	Light	IGKV1-39*01 IGKJ2*01	89.6% (250/279)
**J31**	Heavy	IGHV3-48*01 IGHD3-3*01 IGHJ2*01	91.2% (269/295)
	Light	IGKV1-5*03 IGKJ4*01	97.2% (273/281)
**J47**	Heavy	IGHV4-61*11 IGHD4-11*01 IGHJ4*02	89.2% (264/296)
	Light	IGKV1-5*03 IGKJ4*01	97.9% (275/281)
**J51**	Heavy	IGHV3-23*04 IGHD2-8*01 IGHJ5*02	90.1% (265/294)
	Light	No hits found	
